# Protocol for the AFTERHERNIA Project: patient-reported outcomes of groin and ventral hernia repair

**DOI:** 10.1007/s10029-025-03259-1

**Published:** 2025-01-23

**Authors:** Anders Gram-Hanssen, Jason Joe Baker, Hugin Reistrup, Klaus Kaae Andersen, Jacob Rosenberg

**Affiliations:** 1 Center for Perioperative Optimization, Department of Surgery, Herlev Hospital, University of Copenhagen, Herlev, Denmark; 2 Omicron ApS, Søborg, Denmark

**Keywords:** Hernia, Patient-reported, PROM, Nationwide, Registry, AFTERHERNIA

## Abstract

**Purpose:**

The AFTERHERNIA Project aims to shift the focus of hernia surgery towards patient-reported outcomes by examining the impact of surgical methods and long-term complications on a national level. Groin and ventral hernia repairs are common surgical procedures with significant impact on patient quality of life and healthcare costs. Most large-scale studies focus on clinical outcomes like reoperation and readmission rates, rather than patient-reported outcomes.

**Methods:**

This nationwide survey involves Danish patients who have undergone groin or ventral hernia repair over a ten-year period. Patients will be identified in the Danish National Patient Registry, and they will receive either the Abdominal Hernia-Q or Groin Hernia-Q questionnaire to collect data on patient-reported outcomes. Data from the questionnaire will be linked with clinical and patient-related data from the Danish Hernia Database. The Danish National Patient Registry also contains information on long-term surgical complications. Thereby, it will be possible to link specific perioperative details with patient-reported outcomes and long-term surgical complications.

**Conclusion:**

The AFTERHERNIA Project aims to redefine the understanding of hernia surgery outcomes by emphasizing patient-reported outcomes on a nationwide basis. By capturing a broad spectrum of patient experiences and outcomes, the project expects to inform and possibly transform clinical guidelines and patient care practices.

## Introduction

Hernia surgery accounts for a substantial proportion of all surgical procedures performed globally, affecting quality of life, and imposing substantial costs on healthcare systems worldwide [1]. In Denmark, around 10,000 groin hernia repairs and 5,000 ventral hernia repairs are performed annually [2] incurring considerable expenses for the Danish healthcare system. Postoperative outcomes for Danish patients undergoing hernia repair have previously been investigated extensively on a nationwide basis through the Danish Hernia Database, which has been instrumental in facilitating extensive, population-level research on postoperative outcomes such as reoperation for recurrence and readmission rates. These nationwide studies, along with similar large-scale studies from international registries, have continuously improved the quality of hernia surgery in recent years, both in Denmark and internationally [3, 4]. However, most nationwide studies related to hernia surgery have primarily relied on clinical outcomes rather than patient-reported outcomes. Above all, the outcome of interest in these nationwide studies has been the rate of reoperation for recurrence, which to some extent has guided clinical guidelines and surgical decision-making in the last couple of decades [5, 6]. Considering that the main objective of hernia surgery is to alleviate symptoms and improve quality of life, patient-reported outcomes may represent a more nuanced, conceptually appropriate, and patient-centered outcome of interest for the future [7, 8]. Currently, however, Danish population-level, hernia-related, patient-reported outcome data do not exist.

The AFTERHERNIA Project aims to bridge this evidence gap through its portfolio of studies, each with a distinct focus but unified in their aim to investigate which surgical approaches for groin and ventral hernia repairs yield the best patient-reported outcomes.

## Methods

**Fig. 1:**
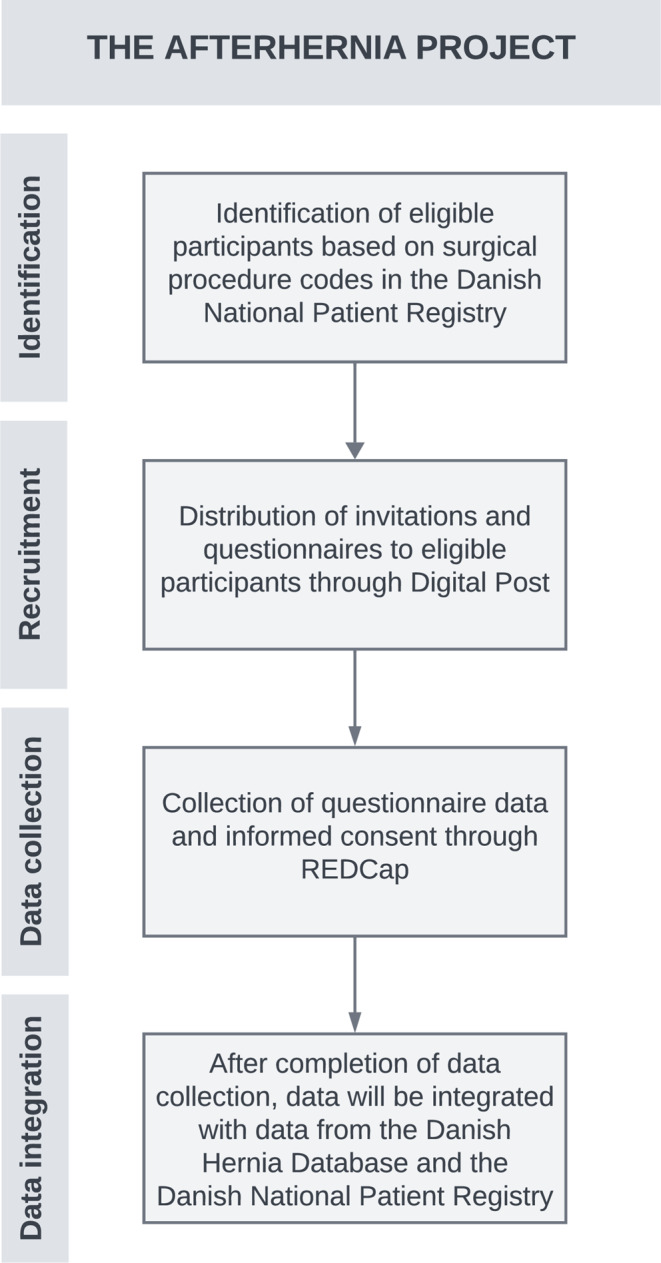
Study design and data flow. REDCap: Research Electronic Data Capture

The AFTERHERNIA Project is a nationwide study including register-based data and surveys among patients in Denmark who have received a groin or ventral hernia repair. The project consists of a portfolio of studies, and the general study design and data flow are illustrated in Fig. 1.

### Participants

Potential participants will be identified based on surgical procedure codes in the Danish National Patient Registry (Tables 1 and 2). Eligible participants will be recruited through Digital Post, which is an online, personal communication service that is provided to all residents in Denmark and is used by approximately 95% of Danish residents ≥ 15 years of age [9]. Eligibility criteria for participation differ between the various studies in the project portfolio, but, in general, patients are eligible if they received a groin or ventral hernia repair in Denmark between 1 January 2014 and 31 March 2024, and if they were ≥ 18 years old at the time of hernia repair. Patients will be excluded if they are deceased, have emigrated, or are exempt from using Digital Post. Patients will also be excluded if they have insufficient knowledge of Danish, or if they are unable to complete the questionnaire due to cognitive or physical impairment, including comorbidities or concurrent surgery that prevent the distinction of symptoms specific to hernias or hernia repair. Patients with non-disclosure of street address will also be excluded as they are protected from contact without prior consent.

**Table 1:** Surgical procedure codes for groin hernia repair

Code		Description	Mesh	Open/Lap
*Inguinal hernia*				
	KJAB00	Inguinal hernia operation	-	Open
	KJAB10	Operation for inguinal hernia with plasty	-	Open
	KJAB11	Laparoscopic inguinal hernia operation	+	Lap
	KJAB20	Inguinal hernia operation with fascia repair	-	Open
	KJAB30	Inguinal hernia operation with synthetic material	+	Open
	KJAB40	Inguinal hernia operation with abdominal wall plasty through laparotomy	-	Open
	KJAB96	Other inguinal hernia operation	?	Open
	KJAB97	Other laparoscopic inguinal hernia operation	+	Lap
*Femoral hernia*				
	KJAC10	Femoral hernia operation	-	Open
	KJAC11	Laparoscopic femoral hernia operation	+	Lap
	KJAC30	Femoral hernia operation with synthetic material	+	Open
	KJAC40	Femoral hernia operation with abdominal wall plasty through laparotomy	-	Open
	KJAC96	Other femoral hernia operation	?	Open
	KJAC97	Other laparoscopic femoral hernia operation	+	Lap

List of surgical procedure codes according to the Nordic Medico-Statistical Classification of Surgical Operations (NOMESCO), used to identify eligible participants in the Danish National Patient Registry. The added “K” in the beginning of the procedure codes means that umbilical hernias are coded as ‘KFAJ’, incisional hernias ‘KJAD’, epigastric hernias ‘KJAE’, other unspecified hernias ‘KJAG’, inguinal hernias ‘KJAB’, and femoral hernias ‘KJAC. Lap: laparoscopy. +: mesh; -: non-mesh;?: unspecified

**Table 2:** Surgical procedure codes for ventral hernia repair

Code		Description	Mesh	Open/Lap
*Umbilical hernia*				
	KJAF10	Operation for umbilical hernia	-	Open
	KJAF11	Laparoscopic operation for umbilical hernia	+	Lap
	KJAF14	Endoscopic retromuscular operation for umbilical hernia	+	Lap
	KJAF14A	Endoscopic retromuscular eTEP operation for umbilical hernia	+	Lap
	KJAF14B	Endoscopic retromuscular eMILOS operation for umbilical hernia	+	Lap
	KJAF20	Operation for umbilical hernia with fascial transplantation	-	Open
	KJAF30	Operation for umbilical hernia with implantation of foreign material	+	Open
	KJAF33	MILOS operation for umbilical hernia	+	Lap
	KJAF96	Other operation for umbilical hernia	?	Open
	KJAF97	Other laparoscopic operation for umbilical hernia	?	Lap
*Epigastric hernia*				
	KJAE10	Operation for epigastric hernia	-	Open
	KJAE11	Laparoscopic operation for epigastric hernia	+	Lap
	KJAE14	Endoscopic retromuscular operation for epigastric hernia	+	Lap
	KJAE14A	Endoscopic retromuscular eTEP operation for epigastric hernia	+	Lap
	KJAE14B	Endoscopic retromuscular eMILOS operation for epigastric hernia	+	Lap
	KJAE30	Operation for epigastric hernia with implantation of foreign material	+	Open
	KJAE33	Minimally invasive retromuscular operation for epigastric hernia (MILOS)	+	Lap
*Incisional hernia*				
	KJAD10	Operation for incisional hernia	-	Open
	KJAD11	Laparoscopic operation for incisional hernia	+	Lap
	KJAD14	Endoscopic retromuscular operation for incisional hernia	+	Lap
	KJAD14A	Endoscopic retromuscular eTEP operation for incisional hernia	+	Lap
	KJAD14B	Endoscopic retromuscular eMILOS operation for incisional hernia	+	Lap
	KJAD20	Operation for incisional hernia with fascial transplantation	-	Open
	KJAD30	Operation for incisional hernia with implantation of foreign material	+	Open
	KJAD33	MILOS operation for incisional hernia	+	Lap
	KJAD96	Other operation for incisional hernia	?	?
*Other unspecified hernia*				
	KJAG00	Operation for other hernia	?	Open
	KJAG01	Laparoscopic operation for other hernia	?	Lap
	KJAG30	Abdominal wall reconstruction with graft	?	Open
	KJAG60	Abdominal wall reconstruction with implantation of foreign material	?	Open

List of surgical procedure codes according to the Nordic Medico-Statistical Classification of Surgical Operations (NOMESCO), used to identify eligible participants in the Danish National Patient Registry. While some of the included codes do not specify the use of mesh, this information will instead be retrieved from the Danish Hernia Database. eTEP: extended totally extraperitoneal repair; eMILOS: extended mini- or less-open sublay operation; Lap: laparoscopy; MILOS: mini- or less-open sublay operation. +: mesh; -: non-mesh;?: unspecified

### Questionnaire

The AFTERHERNIA Project will use the questionnaire Abdominal Hernia-Q (AHQ), which was developed in the United States in 2020 [10]. The AHQ is intended for the assessment of patient-reported outcomes in patients undergoing ventral hernia repair. It features both a pre- and a postoperative form including a total of 24 items with responses scored on 4-point Likert scales. The AHQ covers seven different content domains: expectations, self and others, surgeon and surgical team, sensation, function, appearance, and overall satisfaction [11]. The instrument’s development included extensive patient input and psychometric validation [10–12], and it is likely to be the most thoroughly validated questionnaire for this purpose [13].

The AHQ has recently been translated into Danish and validated for use in a Danish-speaking population (manuscript in preparation). As part of the AFTERHERNIA Project, the AHQ will be adapted and re-validated for patients undergoing groin hernia repair: Groin Hernia-Q (GHQ). A detailed protocol for the questionnaire adaptation and re-validation for groin hernia patients will be published separately. Patients will receive the AHQ after ventral hernia repair and the GHQ after groin hernia repair.

### Registries

The entirety of the AFTERHERNIA Project will be based on data from the Danish National Patient Registry, which offers a comprehensive, nationwide longitudinal record of detailed, routinely collected administrative and clinical data [14]. Since 1977, it has collected information on all public hospital contacts, and since 2003, also from private hospitals and clinics [14]. The Danish National Patient Registry serves as a valuable data source for identifying diseases, examinations, treatments, and surgical procedures [14]. Person-level data from the Danish National Patient Registry will be linked to the Danish Civil Registration System, which holds live electronic records of postal addresses, as well as information on patients who are deceased or have emigrated [15].

All residents in Denmark have a unique and mandatory personal identification number, which is used in all registries and in all contacts with both private and public hospitals, including private practices. The unique personal identification number is also linked to the electronic communication platform Digital Post, which enables survey distribution. All linkage between the included databases and Digital Post will be conducted with the unique personal identification number.

The project will also incorporate data from the Danish Hernia Database, which is a national, surgeon-reported clinical quality registry with mandatory registration of all hernia surgeries performed in Denmark. The Danish Hernia Database has collected data on Danish patients undergoing groin hernia repair since 1998, and on patients undergoing ventral hernia repair since 2007 [16, 17]. In 2022, the national registration rates in the Danish Hernia Database for groin and ventral hernia repairs were 92.8% and 88.5%, respectively [2].

### Data collection and flow

The study population will be defined according to surgical procedure codes (Tables 1 and 2) in the Danish National Patient Registry. The study population will be established by the managing body of the Danish National Patient Registry. Subsequently, invitations to participate will be distributed to patients through Digital Post based on their unique personal identification number, and questionnaires will be completed using Research Electronic Data Capture (REDCap) [18]. Questionnaires will be distributed via unique links, ensuring that each participant receives a personal link to a questionnaire only designated for them, while also ensuring that each participant can only complete the questionnaire once. Participation consent will be obtained simultaneously through REDCap. Multiple reminders will be sent to participants, after which the remaining non-responders will be contacted by phone, if possible. Following informed consent and completion of questionnaires, responses will be integrated with clinical data from the Danish Hernia Database and the Danish National Patient Registry. This integration aims to augment the dataset with information necessary for most of the studies in the portfolio.

### Project portfolio

The first studies in the AFTERHERNIA Project portfolio will focus on the adaptation, translation, and validation of the AHQ and GHQ questionnaires for use throughout the project. This will ensure a solid evidence base for the entirety of the project.

Most subsequent studies will investigate the impact of various perioperative, patient-related, or surgeon-related characteristics on both clinical and patient-reported outcomes. These characteristics of interest include specific types of hernia repair, mesh characteristics, mesh placement, defect closure, specific methods and materials used to fixate these mesh implants, particular patient characteristics, surgeon characteristics, etc. Most studies will be comparative, intending to determine best practice, but a few studies will be descriptive and aim to determine the incidence of specific postoperative complications and their impact on patient-reported outcomes. We will investigate the influence of different mesh materials, mesh weights, mesh placements, mesh fixations, materials and methods for peritoneal closure, and more. Some studies will examine the influence of patient-related factors such as smoking, obesity, and age. Other studies will focus on surgeon-related characteristics, such as the impact of surgical volume and the potential difference in outcomes between surgeries performed by specialists vs. residents.

Lastly, a few studies will take a more meta-methodological approach. Some of these studies will focus on various ways to optimize response rates in large surveys such as this, and other studies will, for instance, investigate the reliability of patient-reported hernia recurrence.

A non-exhaustive list of planned studies in the AFTERHERNIA Project portfolio is available online [19].

### Statistical methods

The statistical methods used will vary based on the specific aims of each study. Generally, analyses will begin with descriptive statistics, summarizing categorical variables (e.g., patient demographics, types of hernia surgery) using frequencies and proportions, and continuous variables (e.g., age, patient-reported outcome scores) using means, medians, standard deviations, or interquartile ranges. These summaries will be presented both for observed data and adjusted for non-response.

To address missing data, including non-responses and incomplete answers, we will employ multiple imputation using chained equations (MICE), assuming data are missing at random (MAR). Sensitivity analyses will explore alternative assumptions, such as data missing not at random (MNAR), using methods like pattern-mixture or selection models. Weighting adjustments based on demographic and clinical characteristics will be used to correct for substantial non-response and ensure representativeness.

Multivariable regression models will assess associations between patient-reported outcomes and various factors. Linear regression will be used for continuous outcomes (e.g., quality of life scores), logistic regression for binary outcomes (e.g., presence of complications), and Cox proportional hazards models for time-to-event outcomes (e.g., time to hernia recurrence). All models will undergo diagnostic checks and be adjusted for non-response and missing data.

To control for type I errors given the exploratory nature of the project, statistical adjustments such as Bonferroni correction or false discovery rate (FDR) methods will be applied. All analyses will be conducted using R, with appropriate packages for imputation, regression modeling, and handling complex survey data.

### Ethical considerations

Approval was obtained from the Capital Region of Denmark authorized under the Danish Data Protection Agency (p-2023-14805). Data will be supplied by the Danish Health Data Authority (FSEID-00006834). Ethical review board approval is neither possible nor required under Danish law. Informed consent will be obtained from all participants. The project poses no risk to participants and adheres to the Declaration of Helsinki [20]. To ensure confidentiality and to prevent any potential stigmatization of specific demographic groups, results will be anonymized, and no individual participants will be identifiable in any published reports.

## Discussion

The AFTERHERNIA Project represents a paradigm shift for population-level hernia research, focusing on patient-reported outcomes to measure surgical success on a national scale. While clinical outcomes have historically dominated, the exclusive focus on such metrics may overlook aspects critical to patients’ quality of life. We aim to close this evidence gap by capturing a broader spectrum of patient experiences.

### Strengths and limitations

The AFTERHERNIA Project integrates detailed registry data from various sources with patient-reported outcome data, which allows for a robust longitudinal assessment of patient outcomes and offers insights into the long-term efficacy of different surgical approaches. The Danish Digital Post service guarantees that this project will reach a broad and representative sample of participants. All data collection will be conducted digitally, which ensures optimal data quality and coverage. The project is also strengthened by having a high external validity by covering all private and public hospitals in Denmark.

However, the project is not without its challenges, and in particular, inadequate response rates pose a risk of non-response bias. To mitigate this, the project plans rigorous follow-up strategies to maximize response rates, and the implementation of weighted analyses to ensure representativeness. Around 5% of Danish resident are exempt from using Digital Post. These are primarily individuals ≥ 75 years old and mostly residents in rural areas of Denmark [9], which presents an unavoidable source of selection bias for the planned studies.

### Clinical implications

The AFTERHERNIA Project has the potential to directly influence clinical guidelines and improve patients’ lives. By providing a clearer picture of which factors lead to improved patient-reported outcomes, healthcare providers can better tailor surgical approaches to individual patient needs, ultimately enhancing patient satisfaction and outcomes.

## Conclusion

The AFTERHERNIA Project will offer significant contributions to both hernia research and clinical practice, emphasizing the importance of patient-centered outcomes in surgical care. By shifting focus towards what truly matters to patients, the project aligns surgical interventions more closely with the goals of enhancing overall quality of life.

## Data Availability

Not applicable.

## References

[1] Ma Q, Jing W, Liu X et al (2023) The global, regional, and national burden and its trends of inguinal, femoral, and abdominal hernia from 1990 to 2019: findings from the 2019 global burden of Disease Study - a cross-sectional study. Int J Surg 109:333–342 37093073 10.1097/JS9.0000000000000217 PMC10389329

[2] Danish Hernia Database Annual reports. https://www.sundhed.dk/content/cms/97/4697_dhdb_aarsrapport_2023_endeligversion.pdf. Accessed 29 Oct 2024

[3] Danish Hernia Database Publications from the Danish Hernia Database. https://www.herniedatabasen.dk/litteraturliste. Accessed 29 Oct 2024

[4] Kyle-Leinhase I, Köckerling F, Jørgensen LN et al (2018) Comparison of hernia registries: the CORE project. Hernia 22:561–575 29307057 10.1007/s10029-017-1724-6 PMC6061062

[5] Rosenberg J, Bisgaard T, Kehlet H et al (2011) Danish hernia database recommendations for the management of inguinal and femoral hernia in adults. Dan Med Bull 58:C4243 21299930

[6] Stabilini C, van Veenendaal N, Aasvang E et al (2023) Update of the international HerniaSurge guidelines for groin hernia management. BJS Open 7:zrad080 37862616 10.1093/bjsopen/zrad080 PMC10588975

[7] Gram-Hanssen A, Tolstrup A, Zetner D et al (2020) Patient-reported outcome measures for patients undergoing inguinal hernia repair. Front Surg 7:17 32373624 10.3389/fsurg.2020.00017 PMC7177003

[8] van Veenendaal N, Poelman MM, van den Heuvel B et al (2021) Patient-reported outcomes after incisional hernia repair. Hernia 25:1677–1684 34338938 10.1007/s10029-021-02477-7 PMC8613099

[9] Agency for Digital Government Statistik om Digital Post. https://digst.dk/it-loesninger/digital-post/om-loesningen/tal-og-statistik/. Accessed 29 Oct 2024

[10] Mauch JT, Enriquez FA, Shea JA et al (2020) The abdominal Hernia-Q: development, psychometric evaluation, and prospective testing. Ann Surg 271:949–957 30601257 10.1097/SLA.0000000000003144

[11] Carney M, Golden K, Weissler J et al (2018) Patient-reported outcomes following ventral hernia repair: designing a qualitative assessment tool. Patient 11:225–234 28856605 10.1007/s40271-017-0275-3

[12] Patel V, Cunning JR, Rios-Diaz AJ et al (2022) Prospective assessment of the abdominal Hernia-Q (AHQ) - patient burden, reliability, and longitudinal assessment of quality of life in hernia repair. Ann Surg 276:1039–1046 33630470 10.1097/SLA.0000000000004713 PMC9645545

[13] Gram-Hanssen A, Tolstrup A, Zetner D et al (2020) Patient-reported outcome measures for inguinal hernia repair are insufficiently validated: a systematic review. Int J Qual Heal Care 32:223–230 10.1093/intqhc/mzaa019 32211859

[14] Schmidt M, Schmidt SAJ, Sandegaard JL et al (2015) The Danish National Patient Registry: a review of content, data quality, and research potential. Clin Epidemiol 7:449–490 26604824 10.2147/CLEP.S91125 PMC4655913

[15] Schmidt M, Pedersen L, Sørensen HT (2014) The Danish Civil Registration System as a tool in epidemiology. Eur J Epidemiol 29:541–549 24965263 10.1007/s10654-014-9930-3

[16] Friis-Andersen H, Bisgaard T (2016) The Danish inguinal hernia database. Clin Epidemiol 8:521–524 27822094 10.2147/CLEP.S99512 PMC5096723

[17] Helgstrand F, Jorgensen LN (2016) The Danish ventral hernia database– a valuable tool for quality assessment and research. Clin Epidemiol 8:719–723 27822119 10.2147/CLEP.S99501 PMC5094577

[18] Harris PA, Taylor R, Thielke R et al (2009) Research electronic data capture (REDCap): a metadata-driven methodology and workflow process for providing translational research informatics support. J Biomed Inf 42:377–381 10.1016/j.jbi.2008.08.010 PMC2700030 18929686

[19] Zenodo Study portfolio for the AFTERHERNIA Project. https://zenodo.org/records/11365247. Accessed 29 Oct 2024

[20] World Medical Association (2013) World Medical Association Declaration of Helsinki: ethical principles for medical research involving human subjects. JAMA 310:2191–2194 24141714 10.1001/jama.2013.281053

